# Establishment of an *in vitro* pingyangmycin-induced mutagenesis system for litchi via embryogenic callus regeneration

**DOI:** 10.3389/fpls.2026.1833403

**Published:** 2026-05-08

**Authors:** Guo Wang, Pengfei Wang, Yaoting Liu, Xueren Cao, Fang Li, Shujun Wang, Huanling Li, Jiabao Wang

**Affiliations:** 1 Environment and Plant Protection Institute, Chinese Academy of Tropical Agricultural Sciences/National Key Laboratory for Tropical Crop Breeding, Haikou, China; 2 Tropical Crops Genetic Resources Institute, Chinese Academy of Tropical Agricultural Sciences, Haikou, China

**Keywords:** embryogenic callus, litchi, mutagenesis, mutant rate, pingyangmycin, somatic mutation

## Abstract

While mutagenesis breeding has been widely applied in major crops, its implementation in tropical horticultural species remains limited. In this study, we established a pingyangmycin-induced mutagenesis system using *in vitro*–cultured embryogenic callus of litchi. Treatment with gradient concentrations of pingyangmycin (0–40 mg/L) revealed an inverse relationship between chemical dosage and cell viability, with somatic embryogenesis completely inhibited at 40 mg/L. Whole-genome resequencing of treated callus identified 307,629 high quality somatic mutations (255,721 SNVs and 51,908 indels), with the 20 mg/L treatment group exhibiting the highest number of mutations. Further resequencing of 40 regenerated mutant lines from the cultivars ‘Feizixiao’ and ‘Lingnan15’ revealed 1,703,663 and 1,281,489 somatic variants, respectively, corresponding to mutation frequencies of 1.8×10^-4^ per site and 1.4×10^-4^ per site, both markedly exceeding typical EMS-induced rates in crops. Transition mutations predominated, accounting for 77% of total single-nucleotide substitutions. Short indels (<3 bp) comprised over 70% of all insertion–deletion events, indicating pingyangmycin’s propensity to induce base substitutions and small indels via oxidative and double-strand break repair pathways. The regenerated mutant plants exhibited substantial leaf morphological variation, confirming effective mutagenic diversification. Collectively, this study provides the first evidence of pingyangmycin’s mutagenic efficacy in litchi, establishes a reliable system for mutant library construction, and delivers valuable genetic resources for functional genomics and molecular breeding applications.

## Introduction

Litchi (*Litchi chinensis Sonn.*) is a fruit of major economic importance in tropical and subtropical regions. By 2025, the litchi cultivation area in China had reached approximately 500,000 hectares, with an annual production of 3.6 million tons (Hu et al., 2025). Despite its substantial industry scale, the litchi breeding technology system remains underdeveloped compared with other fruit crops. Over the past two decades, approximately 63 litchi varieties have been released, yet only 21 have received official authorization, while all of these were derived either from controlled hybridization using known pollen donors or from seedling selection with unknown parentage (Wen et al., 2025). Among them, two traditional cultivars ‘Feizixiao’ and ‘Heiye’ continue to dominate domestic production, together accounting for more than 64% of total yield (Chen et al., 2024). This narrow varietal base has resulted in a homogeneous production structure, limited resilience to biotic and abiotic stresses, and reduced market competitiveness. Consequently, enhancing breeding efficiency and developing novel germplasm resources have become urgent priorities for advancing the high-quality development of the litchi industry.

Mutation is the fundamental source of genetic variation. Among naturally occurring mutations, bud mutation represents a visible and heritable form frequently exploited in fruit tree breeding. Notably, 33.9% of apple cultivars have been developed through bud sport selection (Deng et al., 2019). A genomic analysis of 74 Fuji apple bud mutation lines identified 68,965 somatic variants, shedding light on the clonal evolution of this cultivar (Cai et al., 2024). Artificial mutagenesis has further accelerated crop improvement, leading to the release of more than 3,200 varieties across over 210 plant species (Bado et al., 2015). These mutants not only serve as valuable resources for functional genomics but also provide important intermediate materials for breeding. Fuhui838 is an elite restorer line in the three-line hybrid rice system, developed from the progeny of a cross between Minghui63 and Taiyin1 following Co^60^ γ-ray–induced mutagenesis, while hybrid combinations utilizing this restorer line and its derivatives have achieved cumulative cultivation over 40 million hectares (Deng et al., 2009). Among traditional physical and chemical mutagens, ethyl methane sulfonate (EMS) remains the most widely used. EMS primarily induces G/C→A/T transitions and has been extensively applied to construct large-scale mutant libraries in rice (Hu et al., 2020), maize (Lu et al., 2018), wheat (Wang et al., 2023a), soybean (Sheng et al., 2025), rapeseed (Jhingan et al., 2023), and Chinese cabbage (Sun et al., 2022). However, its high toxicity and environmental persistence limit its use primarily to seed mutagenesis in self-pollinating crops.

Compared with EMS, pingyangmycin demonstrates lower environmental persistence and undergoes rapid degradation, thereby minimizing chemical accumulation and residual toxicity. These properties make pingyangmycin a more sustainable and safer mutagen for plant tissue culture systems. Its low environmental impact and minimal risk to laboratory personnel further underscore its potential as an effective alternative to conventional chemical mutagens such as EMS. To date, pingyangmycin has been successfully applied to generate mutant libraries from non-seed explants in several species, including peanut (Wang et al., 2020), sweet potato (Zhang et al., 2016), and bougainvillea (Chang et al., 2019).

In this study, we established a pingyangmycin-induced mutagenesis system based on *in vitro* embryogenic callus culture of litchi. The optimal pingyangmycin concentration for effective mutagenesis was determined, and mutant libraries were constructed for two major cultivars, ‘Feizixiao’ and ‘Lingnan15’. Whole-genome resequencing of mutant lines revealed that a single pingyangmycin treatment induced an average of 56,682 single-nucleotide variants (SNVs) and 13,744 small insertions or deletions (indels). The resulting mutants displayed extensive genetic and phenotypic variation, providing valuable resources for gene functional analysis and molecular breeding. This system offers a promising approach to enhance genetic improvement and accelerate breeding efficiency in litchi.

## Material and methods

### Callus materials

The experimental materials comprised embryogenic callus tissues of ‘Feizixiao’ and ‘Lingnan15’, maintained through alternating subcultures on alternating subculture between M3 medium (MS + 2,4-D 1 mg·L^-^¹) and M4 medium (MS + 2,4-D 1 mg·L^-^¹ + KT 0.5 mg·L^-^¹ + AgNO_3_ 5 mg·L^-^¹), with one subculture cycle on M3 followed by one subculture cycle on M4 (Wang et al., 2023b). Both media formulations were supplemented with sucrose (30 g·L^-^¹) and agar (7 g·L^-^¹) for *in vitro* preservation.

### Induction treatment of callus tissue

**Figure 1:**
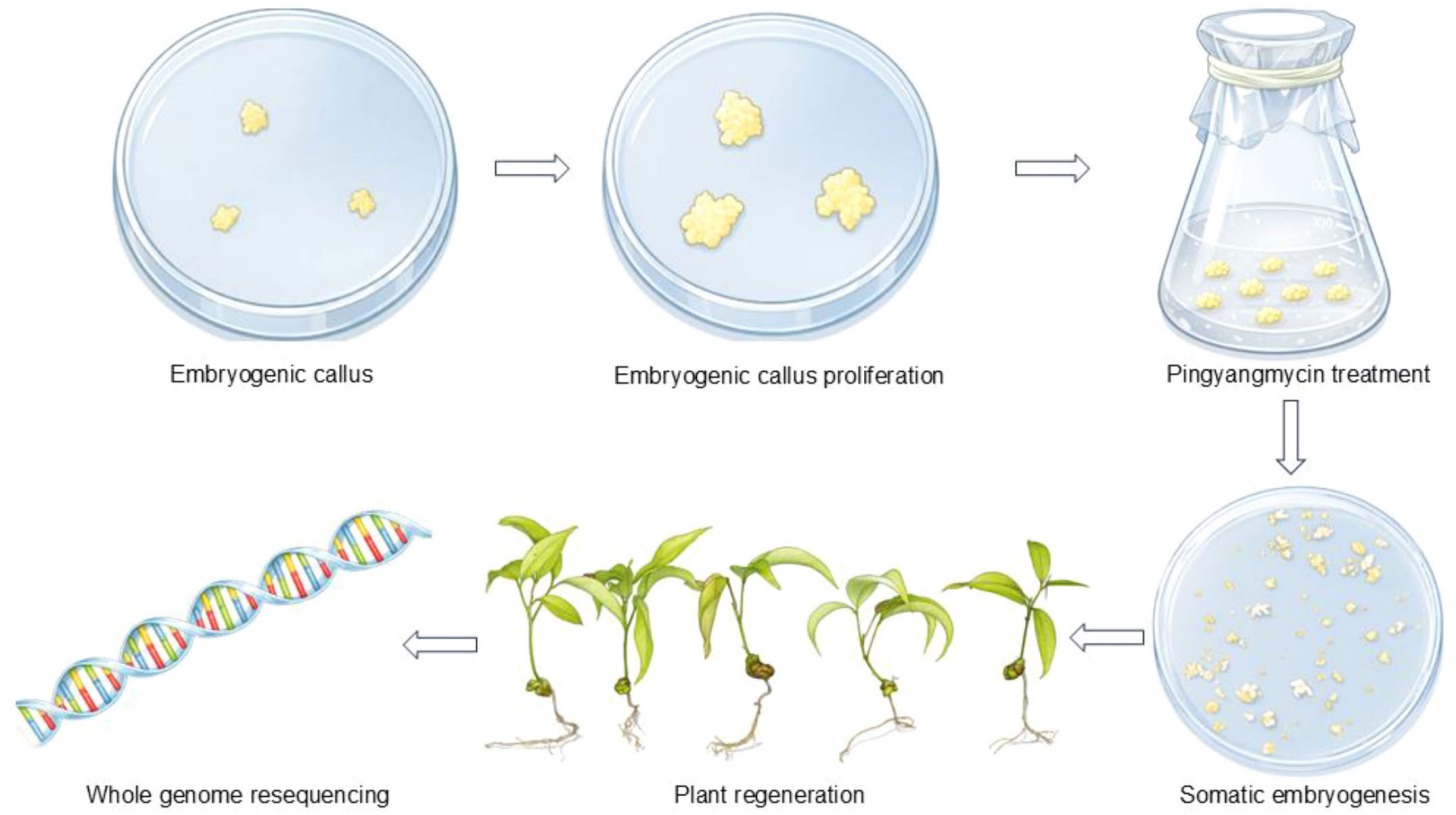
A workflow for pingyangmycin-mediated mutagenesis breeding in litchi.

In this study, ‘Feizixiao’ was initially used as the pilot material to optimize the pingyangmycin treatment conditions, and the optimized protocol was subsequently applied to ‘Lingnan15’. Briefly, embryogenic callus was first proliferated and converted into a stable suspension culture, followed by pingyangmycin treatment under different concentrations and durations. Treated materials were then transferred through proliferation, somatic embryo differentiation, maturation, and germination media to obtain regenerated plants, which were subsequently subjected to phenotypic observation and whole-genome resequencing (**Figure 1**).

Embryogenic callus tissues of *Litchi chinensis* cv. ‘Feizixiao’ were first transferred to proliferation medium (MS supplemented with 1 mg·L^-^¹ 2,4-D, 30 g·L^-^¹ sucrose, and 3.5 g·L^-^¹ gellan gum). After approximately 20 days of culture, 1.5 g of proliferated callus was inoculated into liquid suspension medium (MS + 1 mg·L^-^¹ 2,4-D + 20 g·L^-^¹ sucrose + 150 mg·L^-^¹ myo-inositol + 50 mL·L^-^¹ coconut water) and maintained under continuous agitation. Large aggregates were removed through a 20-mesh sieve, and actively growing suspension cultures were obtained after three subculture cycles (approximately 11 days each) with the removal of non-viable cells. The homogenized suspension was then pre-cultured for 1–2 days before pingyangmycin treatment.

Embryogenic suspension cells were exposed to pingyangmycin at final concentrations of 0, 5, 10, 20, and 40 mg·L^-^¹ for 3 or 7 days to determine the optimal sensitivity threshold and treatment duration. Equal aliquots of suspension cells were transferred into the corresponding media using truncated 1 mL pipette tips to minimize mechanical damage, and the cultures were maintained in darkness at 25 ± 2 °C on an orbital shaker at 120 rpm. Samples were collected at 0, 3, and 7 days to assess single-cell density, cell-cluster density, cell viability, and sedimentation volume in response to pingyangmycin treatment.

### Somatic embryogenesis and germination of callus

Following pingyangmycin treatment, mutagenized embryogenic calli of *Litchi chinensis* cultivars ‘Feizixiao’ and ‘Lingnan15’ were rinsed 4–5 times with sterile distilled water to remove residual mutagen. Rinsed calli were cultured on proliferation medium B for ~25 days. Thereafter, proliferated callus was transferred to somatic embryo differentiation medium (MS supplemented with 0.1 mg·L^-^¹ NAA, 5 mg·L^-^¹ KT, 0.1 g·L^-^¹ myo-inositol, 0.4 g·L^-^¹ LH, 100 mL·L^-^¹ coconut water, 60 g·L^-^¹ sucrose and 10 g·L^-^¹ agar). At 45 days post-transfer, embryogenic responses were assessed by counting milky-white somatic embryos, recording the proportion of dicotyledonous embryos, and documenting embryo morphology.

Differentiated somatic embryos were moved to maturation medium C19 (MS + 0.5 mg·L^-^¹ IAA + 1 mg·L^-^¹ ABA) for approximately 60 days, then transferred to OR4 germination medium (MS + 1 mg·L^-^¹ ornithine) for germination. At 50 days after transfer to OR4, somatic embryogenesis and germination rates of surviving calli were evaluated. Successfully germinated plantlets were acclimatized and subsequently transplanted to the field for further growth and phenotypic assessment.

### DNA extract and whole genome sequencing

Callus tissues treated with five concentrations of pingyangmycin for 7 days (2 g per concentration) and young leaves from forty *in vitro* mutant lines were flash-frozen in liquid nitrogen and stored at –80 °C. Total genomic DNA was extracted from all 45 samples using a modified cetyltrimethylammonium bromide (CTAB) protocol. High-quality DNA was sent to Novogene (Beijing, China) for next-generation sequencing library preparation. Paired-end whole-genome sequencing was performed on the Illumina NovaSeq 6000 platform. In total, 755 Gb of clean data were generated across all samples, corresponding to an average genomic coverage of approximately 35 × per sample.

### Variant identification

The paired-end sequencing reads were trimmed to remove adapters and low-quality bases using fastp with default parameters (Chen et al., 2018). Data quality before and after trimming was evaluated with FastQC (https://www.bioinformatics.babraham.ac.uk/projects/fastqc/). High-quality reads were aligned to the Litchi chinensis reference genome using BWA-MEM, and alignment files were sorted and indexed with SAMtools (Li and Durbin, 2009; Danecek et al., 2021; Li et al., 2024). PCR duplicates were identified and removed using the MarkDuplicates module in GATK. Single-nucleotide variants (SNVs) were initially called using GATK HaplotypeCaller (McKenna et al., 2010).

To obtain high-confidence variants, strict filtering criteria were applied: 1. Quality-based thresholds: QD < 2.0, FS > 60, MQ < 40, SOR > 3.0, MQRankSum < -12.5, and ReadPosRankSum < -8.0. 2. Removal of multi-allelic loci and variants with read depths <50% or >200% of the mean coverage. 3. Retention of somatic mutations showing heterozygous (0/1) genotypes in mutant samples and homozygous reference (0/0) genotypes in wild-type controls.

### Data analysis and visualization

Somatic variants were annotated using SnpEff based on the reference genome annotation (Cingolani et al., 2012; Hu et al., 2022). Downstream statistical analyses and data visualization were performed in the R environment. The ggVennDiagram package was employed to illustrate shared and unique variant sets across treatments, and TBtools was employed to visualize circos charts (Gao et al., 2021; Chen et al., 2023).

## Results

### The impact of pingyangmycin on the growth dynamics of embryonic suspension callus

**Figure 2:**
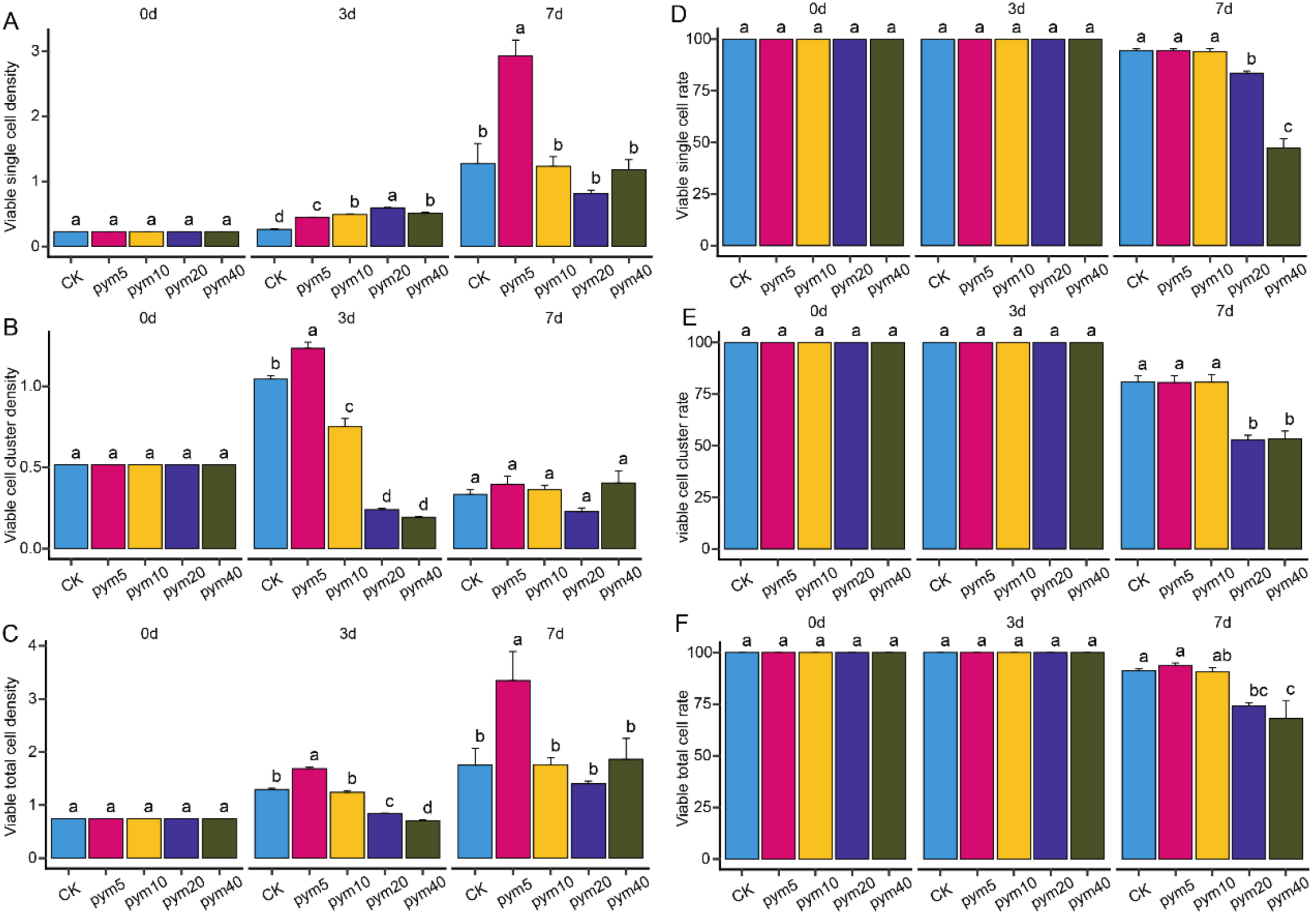
Characteristics of callus tissues treated with five different concentrations of pingyangmycin. Density of viable single cells **(A)**, Density of viable cell clusters **(B)**, Density of viable total cells **(C)**, Rate of viable single cells **(D)**, Rate of viable cell clusters **(E)** and Rate of viable total cells **(F)** in callus tissues treated with five different concentrations of pingyangmycin. Different letters indicate significant difference at *P < 0.05* level via Duncan test.

Litchi embryogenic callus was treated with pingyangmycin at five concentrations (0, 5, 10, 20, and 40 mg/L) to evaluate its effects on cell viability and proliferation. After three days of treatment, the density of viable single cells was highest at 20 mg/L and lowest in the control group. In contrast, viable cell cluster density and total cell density peaked at 5 mg/L and reached their minimum at 40 mg/L (**Figures 2A–C**). Survival rates of viable single cells, viable cell groups, and total cells showed no statistically significant differences among treatments at this stage (**Figures 2D–F**). By the seventh day, the 5 mg/L treatment produced the highest densities of viable single cells and total cells, while the 20 mg/L group exhibited the lowest. Viable cell cluster density did not differ significantly among concentrations, but survival rates for all cell types were markedly reduced under the 40 mg/L treatment. Sedimentation volume increased progressively with treatment duration, with the control group showing the greatest volume and the 20 mg/L group the lowest after seven days (**Supplementary Figure 1**).

### The impact of pingyangmycin on somatic mutations in embryonic suspension callus

**Figure 3:**
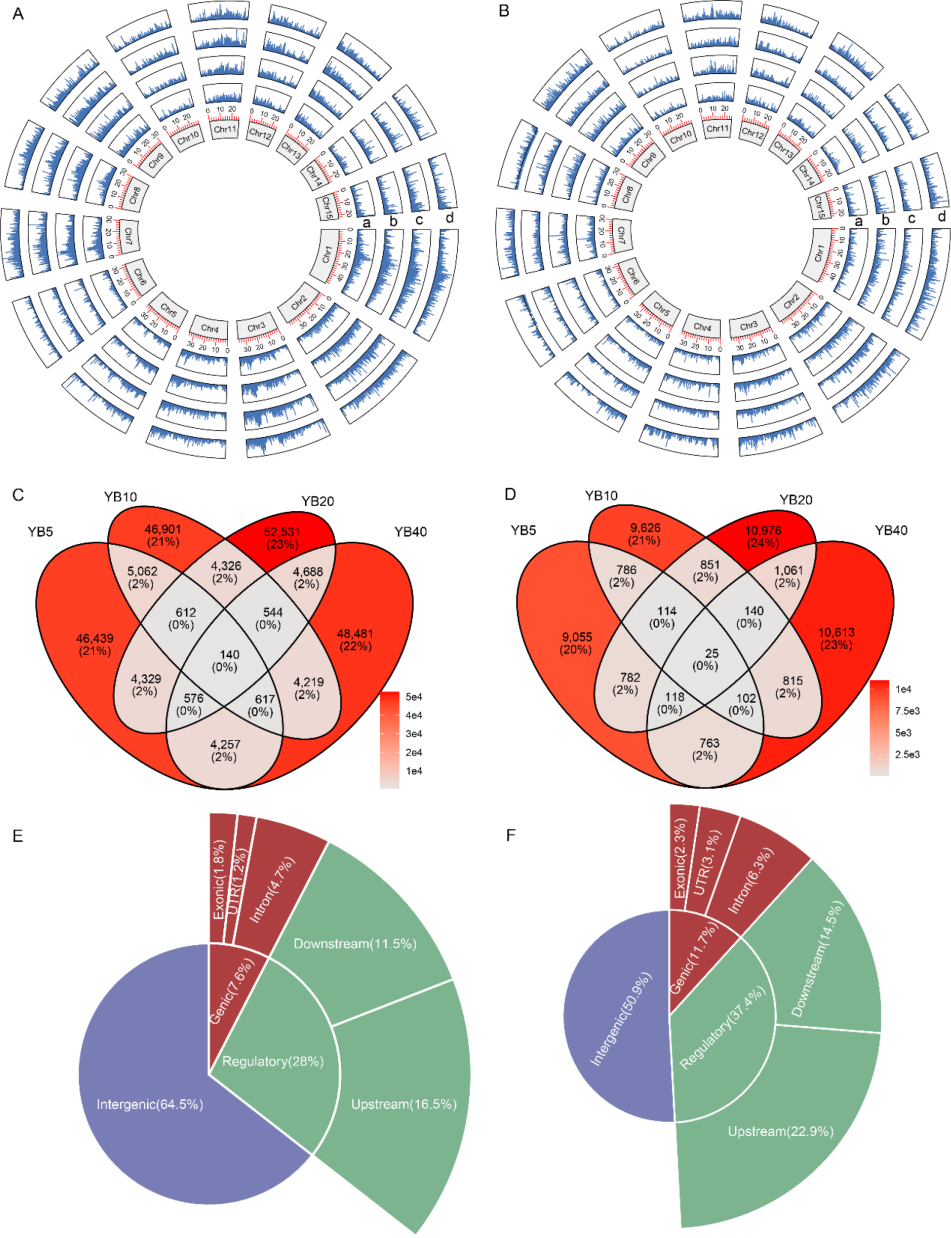
Resequencing analysis of callus tissues treated with five different concentrations of pingyangmycin. **(A)** The distribution of SNVs among the whole genome in four groups (5, 10, 20, and 40 mg/L), a represents 5 mg/L, b represents 10 mg/L, c represents 20mg/L, d represents 40 mg/L. **(B)** The distribution of indels among the whole genome in four groups (5, 10, 20, and 40 mg/L). **(C)** Venn diagram of SNVs in four groups. **(D)** Venn diagram of indels in four groups. **(E)** The genome annotation of all SNVs. **(F)** The genome annotation of all indels.

Embryogenic callus samples treated with five concentrations of pingyangmycin generated a total of 82 Gb of high-quality resequencing data (**Supplementary Table S1**). After read trimming and quality control, 307,629 high-confidence somatic mutations were identified, including 255,721 SNVs and 51,908 indels. Among the treatments, the 5 mg/L group exhibited the lowest mutation count (62,032 SNVs; 11,745 indels), whereas the 20 mg/L group showed the highest (67,746 SNVs; 14,067 indels) (**Figures 3A, B**). Both SNV and indel frequencies increased with rising pingyangmycin concentration, displaying positive correlations with dosage (*R = 0.20*; *R = 0.74*). Pairwise comparisons among treatment groups revealed 5,520–6,431 overlapping somatic SNVs and 1,008–1,344 shared indels (**Figure 3C, D**). Genomic distribution analysis showed that 65.4% of SNVs were located in intergenic regions, 28.0% in gene regulatory elements, and 7.6% within gene bodies. Among the genic SNVs, 228 were predicted to have functional consequences, including stop-gained and start-lost variants (**Figure 3E**). Similarly, 50.9% of indels were intergenic, 37.4% occurred within regulatory regions, and 11.7% were located in coding sequences. Of these, 1,109 indels were classified as potentially disruptive—such as stop-gained and frameshift mutations—likely to alter gene function (**Figure 3F**).

### The impact of pingyangmycin on somatic embryogenesis and subsequent germination

**Figure 4:**
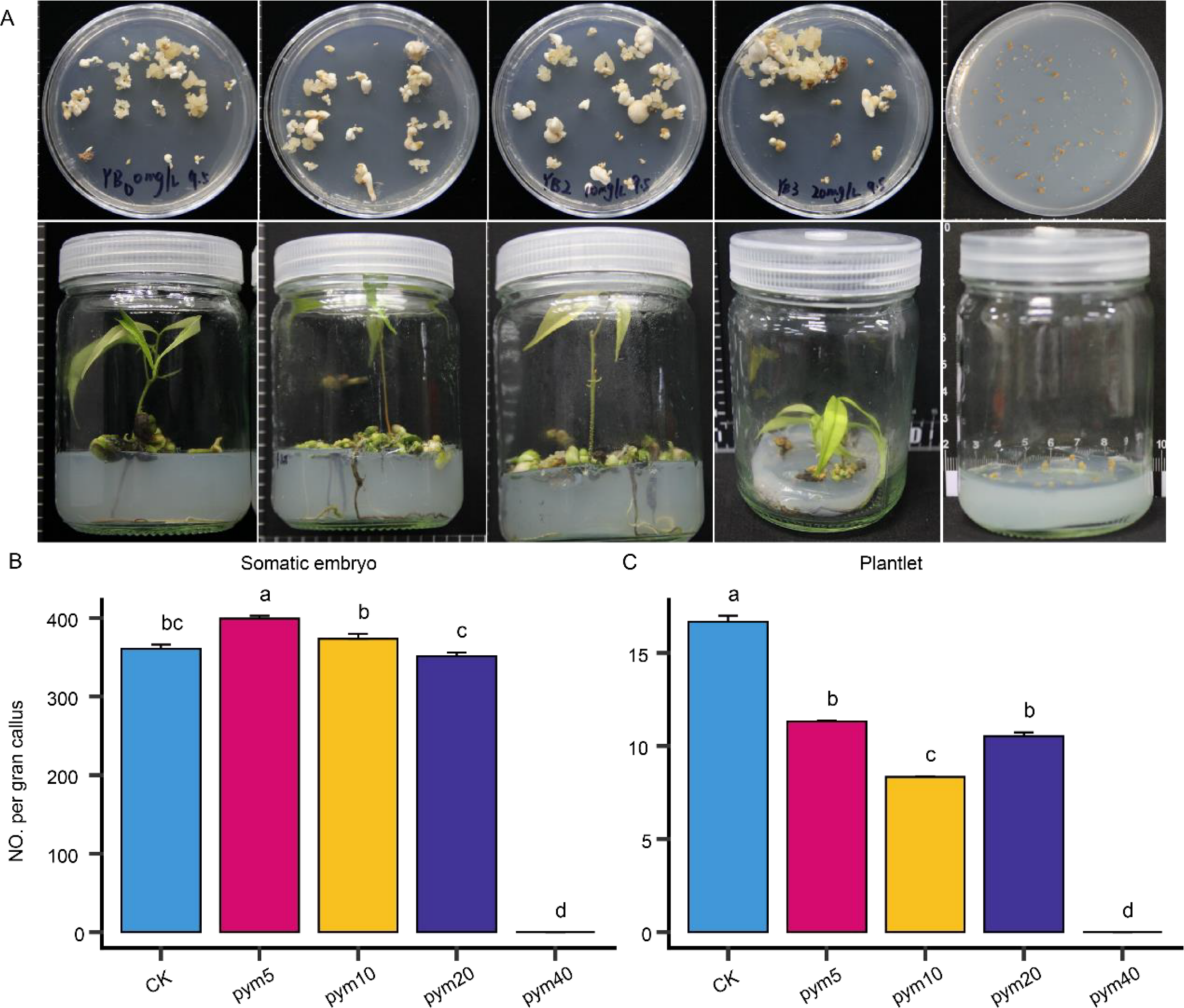
Characteristics of somatic embryogenesis and germination treated with five different concentrations of pingyangmycin. **(A)** Somatic embryogenesis and germination treated with five different concentrations of pingyangmycin. **(B)** The number somatic embryo per gram of callus in five groups. Different letters indicate significant difference at *P < 0.05* level via Duncan test. **(C)** The number of plantlets per gram of callus in five groups.

Following mutagenic treatment, embryogenic suspension callus was induced to form somatic embryos and subsequently regenerated into whole plants (**Figure 4A**). Embryogenic callus exposed to 0–20 mg/L pingyangmycin produced 351–399 somatic embryos per gram of callus and generated 8.3–16.7 regenerated plants per gram of callus (**Figures 4B, C**). The 5 mg/L treatment exhibited the highest embryogenic and regenerative capacity, whereas the 20 mg/L group showed the lowest output. Notably, somatic embryogenesis was completely inhibited at 40 mg/L pingyangmycin.

### Phenotypic diversity of mutant plants from two varieties

**Figure 5:**
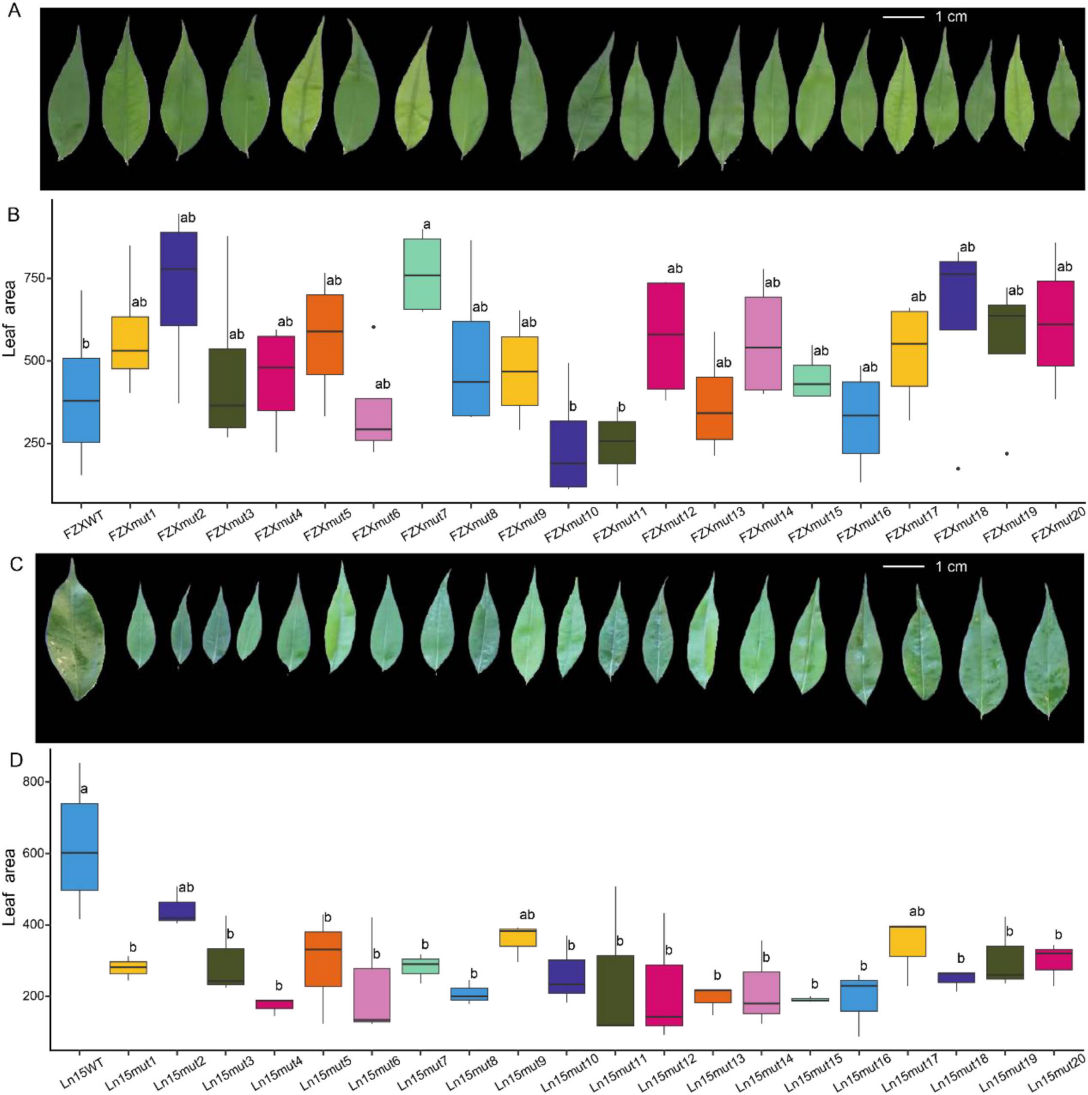
Morphological characteristics of 40 mutant lines. **(A)** Comparison between the 20 mutant lines and the wild type of ‘Feizixiao’. **(B)** Comparison of leaf area between the 20 mutant lines and the wild type of ‘Feizixiao’. Different letters indicate significant difference at *P < 0.05* level via Duncan test. **(C)** Comparison between the 20 mutant lines and the wild type of ‘Lingnan15’. **(D)** Comparison of leaf area between the 20 mutant lines and the wild type of ‘Lingnan15’.

Mutant lines of the cultivars ‘Feizixiao’ and ‘Lingnan15’ exhibited pronounced phenotypic variation in leaf morphology (**Figures 5A, C**). Among the twenty ‘Feizixiao’ mutants, leaf area was generally larger than that of wild-type plants, with FZXmut7 showing the most pronounced enlargement (**Figure 5B**). Most mutants also displayed increased leaf length and perimeter, whereas FZXmut10 and FZXmut11 showed reductions in these traits; FZXmut2 exhibited the greatest overall deviation from the wild type. In contrast, leaf width and aspect ratio did not differ significantly between ‘Feizixiao’ mutants and wild-type plants (**Supplementary Figure 2**). Conversely, the twenty ‘Lingnan15’ mutants predominantly exhibited reduced leaf area relative to wild-type plants, with only Ln15mut2, Ln15mut9, and Ln15mut17 showing no significant differences (**Figure 5D**). Nearly all mutants had markedly decreased leaf length and perimeter, except for Ln15mut11, whose perimeter remained comparable to that of the wild type. Similarly, no significant differences in leaf width or aspect ratio were observed between ‘Lingnan15’ mutants and their wild-type counterparts (**Supplementary Figure 3**).

### Genetic diversity of mutant plants from two varieties

**Figure 6:**
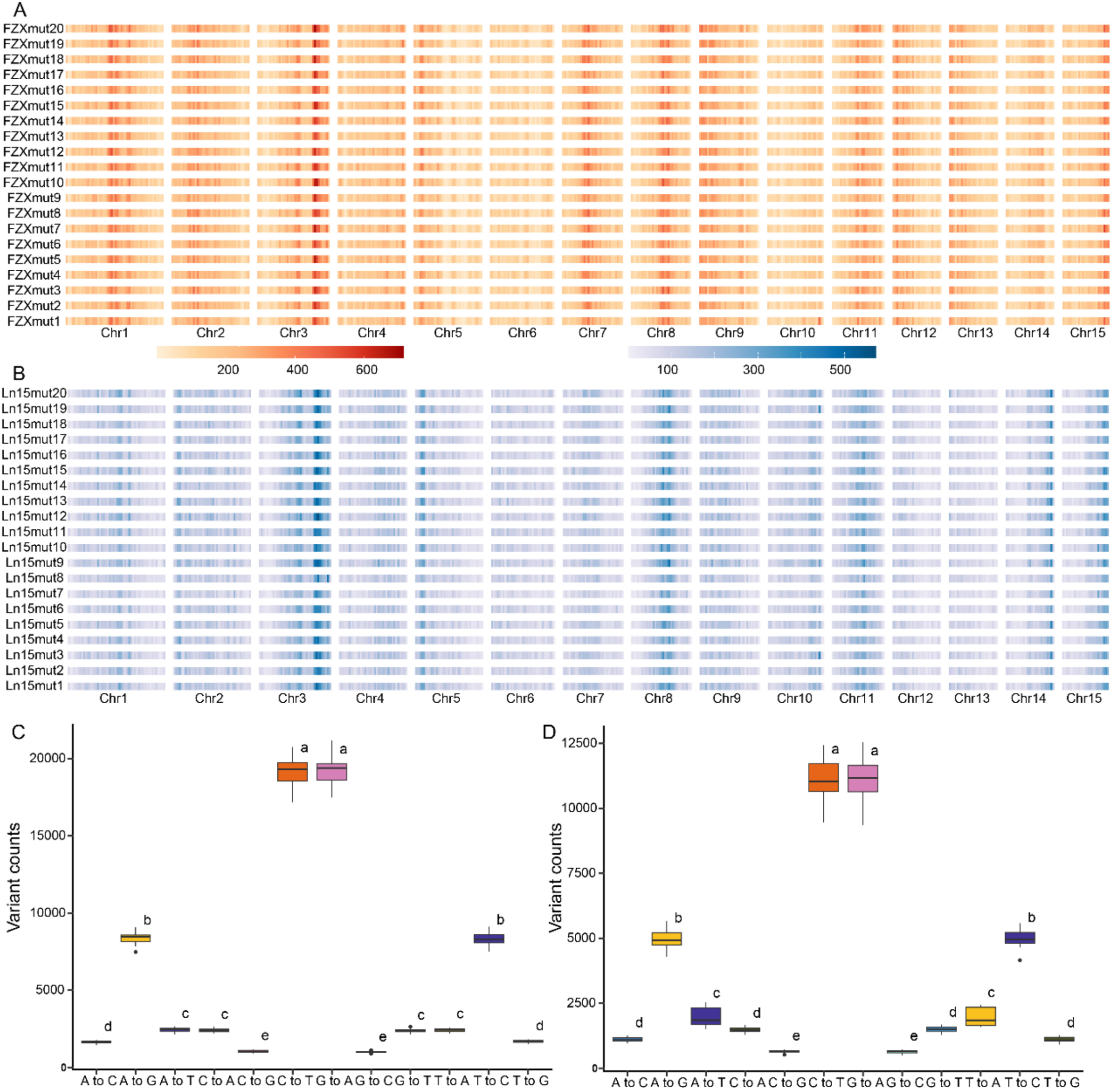
Genetic characteristics of somatic mutations in 40 mutant lines. **(A)** The distribution of somatic mutations among the genome in the 20 mutant lines of ‘Feizixiao’. **(B)** The distribution of somatic mutations among the genome in the 20 mutant lines of ‘Lingnan15’. **(C)** Comparison of different SNV types in the 20 mutant lines of ‘Feizixiao’. Different letters indicate significant difference at *P < 0.05* level via Duncan test. **(D)** Comparison of different SNV types in the 20 mutant lines of ‘Lingnan15’.

Whole-genome resequencing of twenty ‘Feizixiao’ and twenty ‘Lingnan15’ mutant plants produced a total of 672 Gb of high-quality data (**Supplementary Table S1**). After adapter removal and quality control, 1,703,663 high-confidence somatic mutations were identified in ‘Feizixiao’ mutants, comprising 1,399,082 SNVs and 304,581 indels (**Figure 6A**). In comparison, the ‘Lingnan15’ mutants carried 1,281,489 somatic mutations, including 1,006,526 SNVs and 274,963 indels, indicating a substantially higher mutational rate in ‘Feizixiao’ (**Figure 6B**). Within the ‘Feizixiao’ population, FZXmut20 contained the greatest number of SNVs (76,471), while FZXmut11 exhibited the highest indel count (16,224). In contrast, FZXmut13 had the lowest overall mutation load (63,159 SNVs; 14,408 indels). Among ‘Lingnan15’ mutants, Ln15mut12 recorded the highest SNV count (48,012), whereas Ln15mut8 showed the fewest (36,466). The indel frequency was greatest in Ln15mut19 (12,725) and lowest in Ln15mut20 (11,554).

Analysis of nucleotide substitution patterns revealed distinct cultivar-specific biases. In ‘Feizixiao’, G/C→A/T transitions were most prevalent, followed by A/T→G/C transitions, A/T→T/A transversions, C/G→A/T transversions, A/T→C/G transversions, and finally C/G→G/C transversions, which were least common (**Figure 6C**). In contrast, ‘Lingnan15’ exhibited a different hierarchy, with G/C→A/T substitutions being predominant, followed by A/T→G/C transitions, then A/T→T/A, C/G→A/T, and A/T→C/G transversions, with C/G→G/C again occurring at the lowest frequency (**Figure 6D**). Indel size distributions in both cultivars were inversely correlated with indel length (*R* = −0.66; *R* = −0.57). Notably, short indels (< 3 bp) accounted for 70.8% and 71.2% of the total in ‘Feizixiao’ and ‘Lingnan15’, respectively, highlighting a common bias toward small-scale insertion–deletion events induced by Pingyangmycin treatment (**Supplementary Figure 4**).

## Discussion

### 20 mg/L pingyangmycin represents a trade-off between the survival rate and mutation rate of litchi embryogenic callus

The semi-lethal rate is an important parameter in mutation breeding, representing the mutagenic dose at which half of the treated tissues survive. For example, in rice, soaking seeds with 0.5% EMS for 12 hours is considered optimal for maximizing mutant recovery (Unan et al., 2021). In Chinese cabbage, a 0.4% EMS treatment for 16 hours results in a 32.7% survival rate, while in Tartary buckwheat and the wheat cultivar KN9204, soaking with 0.5% and 1% EMS for 12 hours yields survival rates of 56.3% and 41.8%, respectively (Sun et al., 2022; Wang et al., 2023a; Li et al., 2025). Bleomycin, a low-toxicity and effective chemical mutagen, operates through metal ion- and oxygen-dependent oxidative DNA damage, preferentially binding or cleaving DNA at guanine-rich regions, while its main active component is pingyangmycin (Bailly et al., 1997). Previous studies have demonstrated the applicability of pingyangmycin in plant tissue culture: in peanut cultivar ‘Huayu20’, treatment with 4 mg/L pingyangmycin for four weeks resulted in approximately 50% somatic embryo formation; in sweet potato cultivar ‘Yanshu25’, exposure to 3 mg/L pingyangmycin produced three distinct mutant lines; and in bougainvillea cutting mutagenesis, pingyangmycin concentrations up to 1% had no significant impact on cutting survival (Zhang et al., 2016; Chang et al., 2019; Wang et al., 2020).

In this study, we applied pingyangmycin at five concentration gradients (0, 5, 10, 20, and 40 mg/L) to litchi embryogenic callus. After seven days of treatment, total cell survival was inversely correlated with pingyangmycin concentration. Notably, even at 40 mg/L, survival remained as high as 68%, indicating that the conventional semi-lethal dose standard used for EMS-based mutagenesis was not applicable. Whole-genome resequencing of callus samples revealed that the number of somatic mutations increased with pingyangmycin concentration but peaked at 20 mg/L before declining at 40 mg/L. This reduction at higher concentration likely reflects decreased cell viability, consistent with observations during somatic embryogenesis and germination, where nearly no normal embryos or intact plants were regenerated under 40 mg/L treatment. Collectively, these findings indicate that a 20 mg/L pingyangmycin concentration provides an optimal balance between mutation frequency and cell survival in litchi embryogenic callus. This condition forms the foundation for establishing an efficient pingyangmycin-induced mutagenesis system for litchi.

### A high induction rate, frequent transition mutations, and abundant short indels are characteristic mutational features of pingyangmycin treatment

In plants, the spontaneous mutation rate is generally around 10^-8^ per site per generation, while the commonly accepted rate for *Arabidopsis thaliana* is approximately 7×10^-9^ per site per generation (Ossowski et al., 2010). In contrast, the mutation rate in EMS-induced mutants is substantially higher. For example, the M2 generation of EMS-treated Chinese cabbage exhibits a mutation rate of 2.5×10^-5^, while EMS-induced M2 and later generations of Tartary buckwheat and M7 generation of soybean cultivar ‘Tianlong 1’ show mutation rates of 1.9×10^-5^ and 1.7×10^-5^, respectively (Sun et al., 2022; Li et al., 2025; Sheng et al., 2025).

In the present study, the mutation rate of pingyangmycin-induced M0 mutant lines was 1.8×10^-4^ per site in ‘Feizixiao’ and 1.4×10^-4^ per site in ‘Lingnan15’, both markedly higher than those typically induced by EMS. EMS is known to alkylate guanine, resulting in G/C→A/T transition mutations during replication (Sega, 1984). Consistently, resequencing and variant analysis of 40 pingyangmycin-induced mutant lines revealed that G/C→A/T transitions accounted for 53.3% of all SNVs, while A/T→G/C transitions constituted 23.7%, yielding a transition/transversion (Ti/Tv) ratio of 3.3. For comparison, the Ti/Tv ratio of natural mutations in Arabidopsis thaliana is approximately 2.3, indicating that pingyangmycin-induced mutagenesis is biased toward transition-type substitutions (Greene et al., 2003). Additionally, resequencing analysis showed that short indels of less than 3 bp comprised 71% of all detected indels. This suggests that pingyangmycin induces mutagenesis through two complementary mechanisms: (1) promoting base substitution bias toward transitions via oxidative DNA damage during replication or repair, and (2) generating abundant short indels through DNA double-strand breaks followed by repair through non-homologous end joining (NHEJ) or microhomology-mediated end joining (MMEJ) pathways.

### A pingyangmycin-based mutagenesis breeding system significantly enhances the efficiency of genetic breeding in litchi

‘Feizixiao’ is the predominant cultivar in current litchi production, valued for its ease of flowering and high yield. However, the disadvantages of small fruit size, uneven pericarp coloration, and concentrated ripening period limit the market appeal (Zhao et al., 2025). As a perennial evergreen fruit tree, litchi exhibits a prolonged juvenile phase, typically requiring more than five years from hybrid seed to flowering (Zhang et al., 2025). Moreover, as a highly heterozygous and naturally outcrossing species, litchi breeding traditionally relies on elite cultivars as parents. Yet, hybrid progeny often displays extensive trait segregation, greatly increasing the cost and time of conventional breeding. Mutagenesis breeding offers an efficient approach to accelerate genetic improvement in plants. For instance, the rice variety Zhefu802, developed from Co^60^ γ-ray–induced mutagenesis of Simei2, exhibits early maturity and high yield, with cumulative cultivation exceeding 100 million mu as an early-season indica rice (https://www.ricedata.cn/variety/varis/600505.htm). Similarly, the wheat cultivar Luyuan502, derived from the hybrid progeny of the space-mutated material 9940168 and the elite line Jimai19, has been designated as a key promoted variety by both national and provincial authorities for several consecutive years (Li et al., 2013). In this study, elite cultivars such as ‘Feizixiao’ and ‘Lingnan15’ were used as basic materials to establish a pingyangmycin-induced mutagenesis system based on embryogenic callus regeneration, while phenotypic diversity of leaves was observed even in the seeding stage. This approach enabled the rapid generation of numerous litchi variants exhibiting diverse and improved traits, providing a promising strategy to enhance the efficiency of litchi genetic improvement and promote the high-quality development of the litchi industry.

## Data Availability

All raw reads generated for the individuals in the study have been deposited in the National Genomics Data Center under BioProject PRJCA051057.

## References

[B1] Bado S. Forster B. P. Nielen S. Ali A. M. Lagoda P. J. L. Till B. J. . (2015). Plant mutation breeding: Current progress and future assessment. Plant Breed. Rev. 39, 23–88. doi: 10.1002/9781119107743.ch02

[B2] Bailly C. Kénani A. Waring M. J. Liu Y. Tong L. Chen X. L. (1997). Altered cleavage of DNA sequences by bleomycin and its deglycosylated derivative in the presence of actinomycin. Nucleic Acids Res. 25, 1516–1522. doi: 10.1093/nar/25.8.1516. PMID: 9106360 PMC146634

[B3] Cai Y. D. Gao X. H. Mao J. P. Yang G. S. . (2024). Genome sequencing of 'fuji' apple clonal varieties reveals genetic mechanism of the spur-type morphology. Nat. Commun. 15, 10082. doi: 10.1038/s41467-024-54428-2. PMID: 39572540 PMC11582680

[B4] Chang S. X. Huang S. R. Xu S. S. Wang X. Zeng Z. Xu J. . (2019). Chemical mutagenesis for the cuttings of bougainvillea glabra 'mrs. Eva' and b. × buttiana 'miss manila'. Chin. J. Trop. Crops 2, 238–246. doi: 10.3969/j.issn.1000-2561.2019.02.005

[B5] Chen C. Wu Y. Li J. Ou S. Pan W. Peng X. . (2023). Tbtools-ii: A "one for all, all for one" bioinformatics platform for biological big-data mining. Mol. Plant 16, 1733–1742. doi: 10.1016/j.molp.2023.09.010. PMID: 37740491

[B6] Chen H. Yang S. Su Z. Gu J. (2024). Analysis of the national litchi production in 2024 and management suggestions. China Trop. Agric. 3, 8–20.

[B7] Chen S. Zhou Y. Chen Y. Coon M. Nguyen T. Wang L. . (2018). Fastp: An ultra-fast all-in-one fastq preprocessor. Bioinformatics 34, i884–i890. doi: 10.1093/bioinformatics/bty560. PMID: 30423086 PMC6129281

[B8] Cingolani P. Platts A. Wang L. L. Marshall J. Ohan V. Pollard M. O. . (2012). A program for annotating and predicting the effects of single nucleotide polymorphisms, snpeff: Snps in the genome of drosophila melanogaster strain w1118; iso-2; iso-3. fly 6, 80–92. doi: 10.4161/fly.19695. PMID: 22728672 PMC3679285

[B9] Danecek P. Bonfield J. K. Liddle J. Feng L. Yang X. Ma A. . (2021). Twelve years of samtools and bcftools. GigaScience 10, giab008. doi: 10.1093/gigascience/giab008. PMID: 33590861 PMC7931819

[B10] Deng D. Chen H. Deng W. Zhang S. Zhang Z. Cong P. . (2009). Development of a indica rice restorer line fuhui838 and its derivative lines with strong restoring ability and their utilization. Acta Agric. Nucleat. Sin. 23, 175–179.

[B11] Deng X. Wang L. Li S. Henikoff J. G. Till B. J. Reynolds S. H. . (2019). Retrospection and prospect of fruit breeding for last four decades in China. J. Fruit Sci. 36, 514–520. doi: 10.13925/j.cnki.gsxb.20190094

[B12] Gao C. H. Yu G. Cai P. Wang J. Salojarvi J. Liu C. (2021). Ggvenndiagram: An intuitive, easy-to-use, and highly customizable r package to generate venn diagram. Front. Genet. 12, 706907. doi: 10.3389/fgene.2021.706907. PMID: 34557218 PMC8452859

[B13] Greene E. A. Codomo C. A. Taylor N. E. Wu H. Han Z. Xiao J. . (2003). Spectrum of chemically induced mutations from a large-scale reverse-genetic screen in arabidopsis. Genetics 164, 731–740. doi: 10.1093/genetics/164.2.731. PMID: 12807792 PMC1462604

[B14] Hu G. Feng J. Xiang X. Dreyer F. Abbadi A. Beckmann K. . (2022). Two divergent haplotypes from a highly heterozygous lychee genome suggest independent domestication events for early and late-maturing cultivars. Nat. Genet. 54, 73–83. doi: 10.1038/s41588-021-00971-3. PMID: 34980919 PMC8755541

[B15] Hu G. B. Yang S. N. Qi W. E. Zhang F. Y. Sun M. Z. (2025). Analysis of China's litchi production in 2025 and management recommendations. China Trop. Agric 2, 8–16. doi: 10.1201/9781003305033-7

[B16] Hu W. Zhou T. Hu G. Jian Y. L. Ran B. Cheng Y. Z. . (2020). An ethyl methanesulfonate-induced neutral mutant-bridging method efficiently identifies spontaneously mutated genes in rice. Plant J. 104, 1129–1141. doi: 10.1111/tpj.14969. PMID: 32808346

[B17] Jhingan S. Kumar A. Harloff H. J. Yang Q. Chai Z. Chen R. . (2023). Direct access to millions of mutations by whole genome sequencing of an oilseed rape mutant population. Plant J. 113, 866–880. doi: 10.1111/tpj.16079. PMID: 36575585

[B18] Li H. Durbin R. Sivachenko A. Cibulskis K. Kernytsky A. (2009). Fast and accurate short read alignment with burrows-wheeler transform. Bioinformatics 25, 1754–1760. doi: 10.1093/bioinformatics/btp324. PMID: 19451168 PMC2705234

[B19] Li H. Y. Lv Q. Y. Shi T. X. Warthmann N. Clark R. M. Shaw R. G. . (2025). The complete reference genome of tartary buckwheat and its mutation library provide important resources for genetic studies and breeding. Cell Rep. 44. doi: 10.1016/j.celrep.2025.115621. PMID: 40327509

[B20] Li J. Chen C. Zeng Z. Wu F. Feng J. Liu B. . (2024). Sapbase: A central portal for functional and comparative genomics of sapindaceae species. J. Integr. Plant Biol. 66, 1561–1570. doi: 10.1111/jipb.13680. PMID: 38804840

[B21] Li X. H. Li P. Gao G. Q. Wang S. Zhang X. M. Zhang K. . (2013). Characteristics of new wheat cultivar luyuan 502 with high yield and wide adaption and its breeding strategies. Shandong. Agric. Sci. 45, 32–34.

[B22] Lu X. Liu J. Ren W. Jian Y. L. Ran B. Cheng Y. Z. . (2018). Gene-indexed mutations in maize. Mol. Plant 11, 496–504. doi: 10.1016/j.molp.2017.11.013. PMID: 29223623

[B23] McKenna A. Hanna M. Banks E. Yang Q. Chai Z. Chen R. . (2010). The genome analysis toolkit: A mapreduce framework for analyzing next-generation DNA sequencing data. Genome Res. 20, 1297–1303. doi: 10.1101/gr.107524.110. PMID: 20644199 PMC2928508

[B24] Ossowski S. Schneeberger K. Lucas-Lledo J. I. Sivachenko A. Cibulskis K. Kernytsky A. . (2010). The rate and molecular spectrum of spontaneous mutations in arabidopsis thaliana. Science 327, 92–94. doi: 10.1126/science.1180677. PMID: 20044577 PMC3878865

[B25] Sega G. A. Liu H. L. Yan Q. Warthmann N. Clark R. M. Shaw R. G. (1984). A review of the genetic effects of ethyl methanesulfonate. Mutat. Res. 134, 113–142. doi: 10.1016/0165-1110(84)90007-1. PMID: 6390190

[B26] Sheng Y. Huang Y. Jin Z. Wang X. Liu C. Zhang J. . (2025). Assembly of a high-quality reference genome and characterization of a chemical-mutagenized library of an elite soybean cultivar tianlong 1. J. Genet. Genomics 53, 458–466. doi: 10.1016/j.jgg.2025.08.006. PMID: 40848895

[B27] Sun X. X. Li X. Lu Y. Wang S. Zhang X. M. Zhang K. . (2022). Construction of a high-density mutant population of chinese cabbage facilitates the genetic dissection of agronomic traits. Mol. Plant 15, 913–924. doi: 10.1016/j.molp.2022.02.006. PMID: 35150930

[B28] Unan R. Deligoz I. Al-Khatib K. Mennan H. (2021). Protocol for ethyl methanesulphonate (ems) mutagenesis application in rice. Open Res. Eur. 1, 19. doi: 10.12688/openreseurope.13317.3. PMID: 37645151 PMC10446052

[B29] Wang D. Z. Li Y. P. Wang H. J. Xu Y. X. Yang Y. M. Zhou Y. X. . (2023a). Boosting wheat functional genomics via an indexed ems mutant library of kn9204. Plant Commun. 4, 100593. doi: 10.1016/j.xplc.2023.100593. PMID: 36945776 PMC10363553

[B30] Wang G. Liu Y. Gao Z. Li H. Wang J. . (2023b). Effects of amino acids on callus proliferation and somatic embryogenesis in litchi chinensis cv.'Feizixiao'. Horticulturae 9, 1311. doi: 10.3390/horticulturae9121311. PMID: 30654563

[B31] Wang J. S. Shi L. Liu Y. Zhao M. X. Wang X. Qiao L. X. . (2020). Development of peanut varieties with high oil content by mutagenesis and screening. J. Integr. Agr. 19, 2974–2982. doi: 10.1016/s2095-3119(20)63182-3

[B32] Wen Y. J. Tang Z. X. Shi F. C. Liu H. L. Yan Q. . (2025). Research on the protection status and development strategies for new varieties of lychee and longan in China. Guangdong. Agric. Sci. 52, 120–132. doi: 10.16768/j.issn.1004-874X.2025.04.012

[B33] Zhang D. Wang W. Li G. Sui J. Qiao L. Wang J. . (2016). Chemistry mutagenesis *in vitro* and characteristics of mutants in sweet potato. Plant Physiol. J. 52, 343–348. doi: 10.13592/j.cnki.ppj.2015.0698

[B34] Zhang L. Wang P. Li F. Xu L. Zhao J. Fu J. . (2025). Litchi40k v1. 0: A cost-effective, flexible, and versatile liquid snp chip for genetic analysis and digitalization of germplasm resources in litchi. Hortic. Res. 12, uhaf038. doi: 10.1093/hr/uhaf038. PMID: 40236734 PMC11997437

[B35] Zhao M. Huang Q. Zhang B. Guo W. Lv G. Wang D. . (2025). Histological and transcriptomic analysis reveals the cell number and plant hormone related to fruit size in litchi chinensis sonn. Sci. Hortic. 341, 114007. doi: 10.1016/j.scienta.2025.114007. PMID: 38826717

