# Supplementary material for: Establishment of an in vitro pingyangmycin-induced mutagenesis system for litchi via embryogenic callus regeneration
Source: Front Plant Sci. 2026 May 8;17:1833403. doi: 10.3389/fpls.2026.1833403 (PMC13194384; doi:10.3389/fpls.2026.1833403)
Supplement: Supplementary file 1 [file DataSheet1.docx]

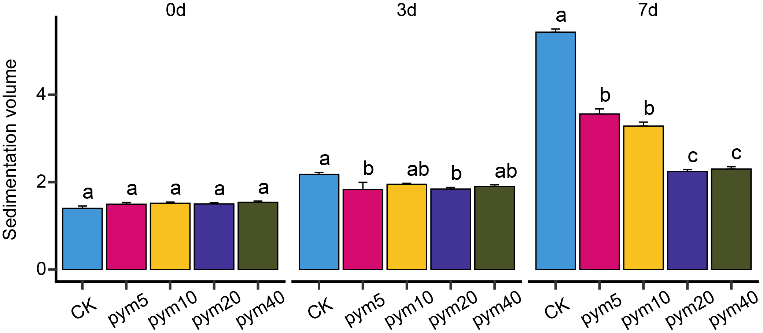


**Supplementary Fig. 1 Sedimentation volume in callus tissues treated with five different concentrations of pingyangmycin**

Different letters indicate significant difference at *P < 0.05* level via Duncan test.


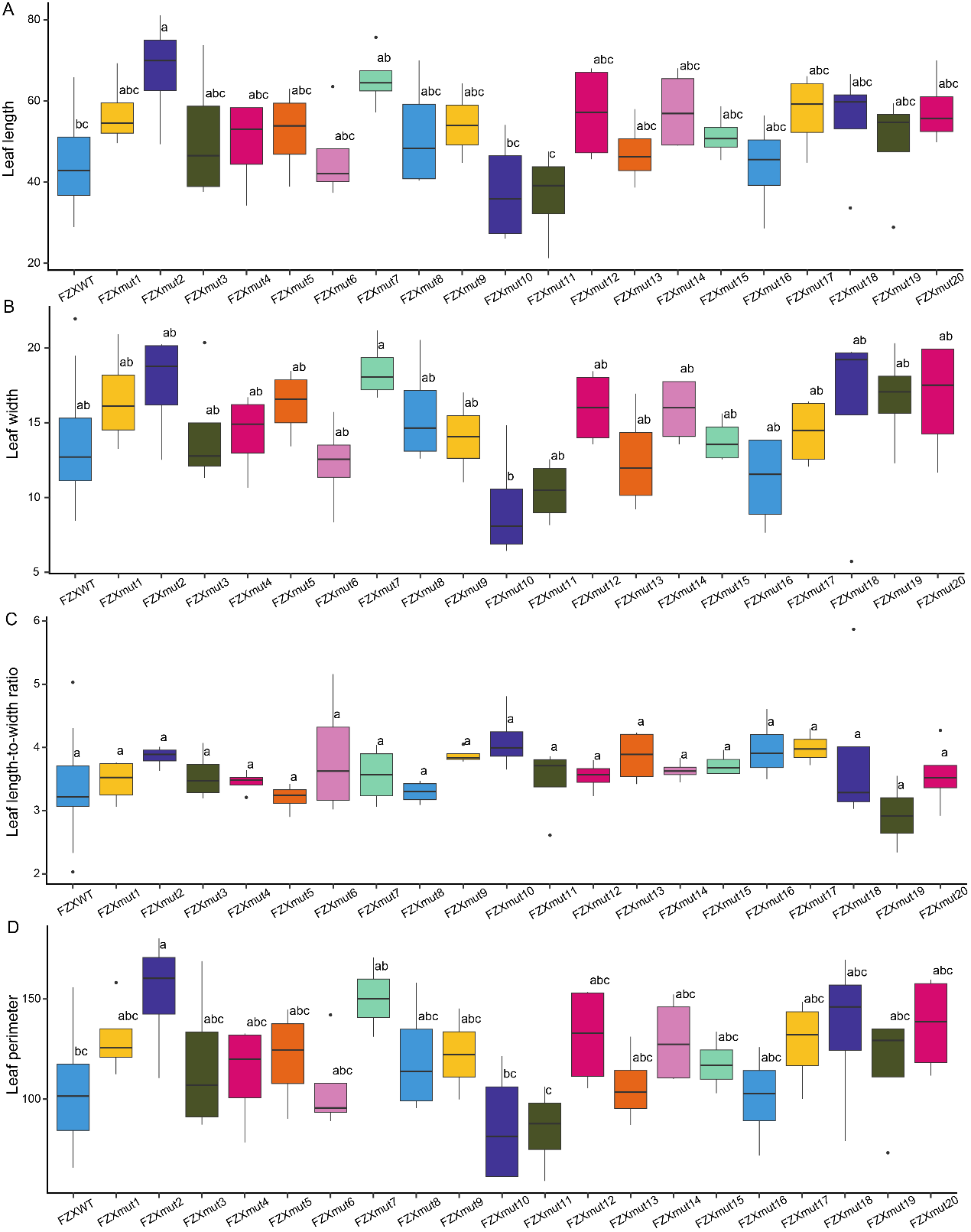


**Supplementary Fig. 2 Morphological characteristics of 20 mutant lines of ‘Feizixiao’**

The comparison of Leaf length (A), Leaf width (B), Leaf length-to-width ratio (C), Leaf perimeter (D) between the 20 mutant lines and the wild type of ‘Feizixiao’. Different letters indicate significant difference at *P < 0.05* level via Duncan test.


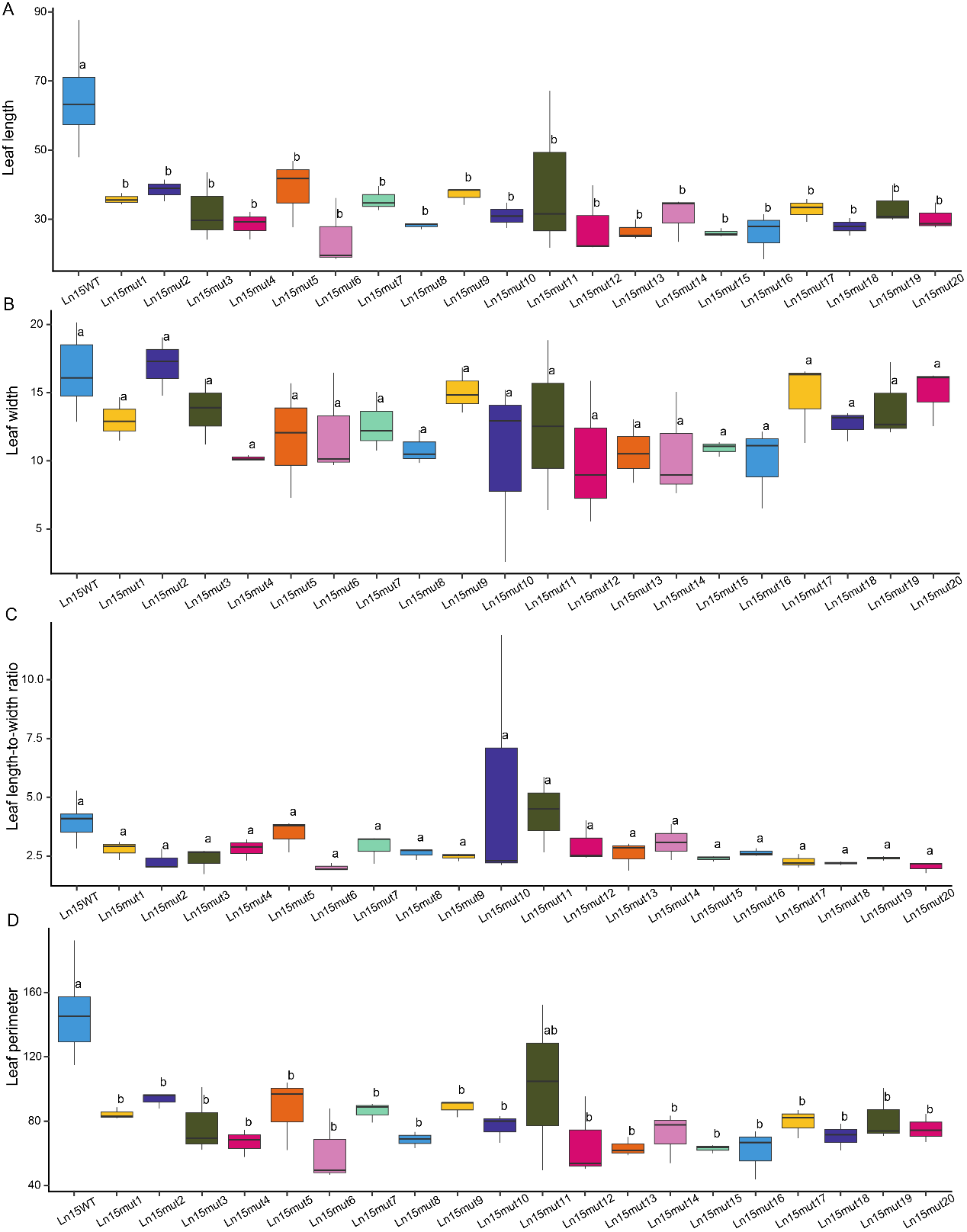


**Supplementary Fig. 3 Morphological characteristics of 20 mutant lines of ‘Lingnan15’**

The comparison of Leaf length (A), Leaf width (B), Leaf length-to-width ratio (C), Leaf perimeter (D) between the 20 mutant lines and the wild type of ‘Lingnan15’. Different letters indicate significant difference at *P < 0.05* level via Duncan test.


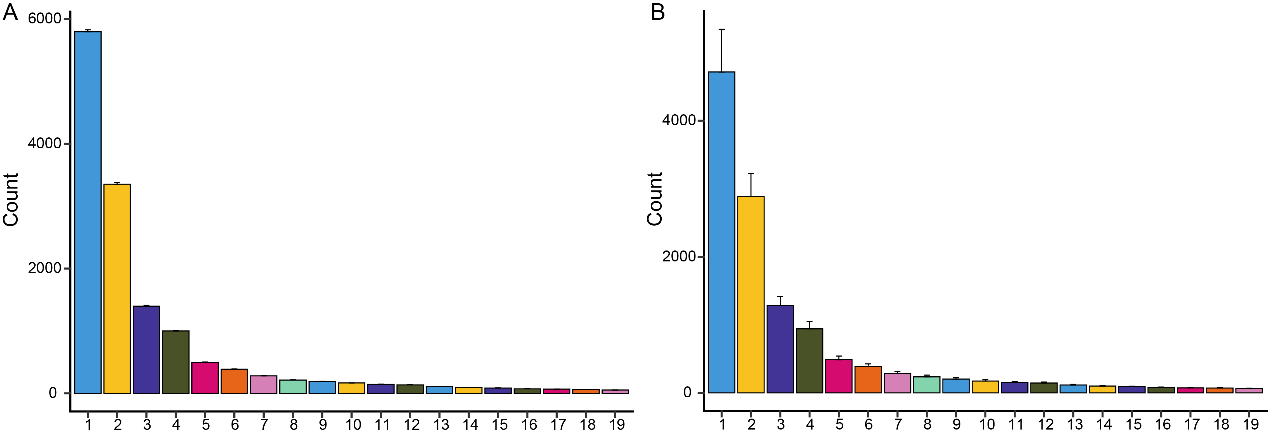


**Supplementary Fig. 4 The distribution of indel size in the 20 mutant lines of ‘Feizixiao’ (A) and 20 mutant lines of ‘Lingnan15’(B)**
