# Supplementary material for: Establishment of an in vitro pingyangmycin-induced mutagenesis system for litchi via embryogenic callus regeneration
Source: Front Plant Sci. 2026 May 8;17:1833403. doi: 10.3389/fpls.2026.1833403 (PMC13194384; doi:10.3389/fpls.2026.1833403)
Supplement: Supplementary file 2 [file Table1.docx]

**TableS1 Statistical analysis of resequencing of 45 samples**

| **Sample** | **reads** | **bases** | **Q20** | **Q30** | **Covrage** |
| --- | --- | --- | --- | --- | --- |
| YB0 | 54894602 | 16451107733 | 97.3 | 92.5 | 34.0 |
| YB5 | 54905938 | 16457442967 | 97.2 | 92.3 | 34.0 |
| YB10 | 55633044 | 16672048595 | 96.9 | 91.5 | 34.5 |
| YB20 | 57264756 | 17158889217 | 96.8 | 91.4 | 35.5 |
| YB40 | 51312021 | 15377885171 | 97.1 | 92.0 | 31.8 |
| FZXmut1 | 56851061 | 17040331440 | 99.4 | 97.8 | 35.2 |
| FZXmut10 | 59615040 | 17869462182 | 99.4 | 97.7 | 37.0 |
| FZXmut11 | 59591988 | 17861241386 | 99.4 | 97.7 | 36.9 |
| FZXmut12 | 56606282 | 16962597270 | 99.4 | 97.7 | 35.1 |
| FZXmut13 | 56820665 | 17030439566 | 99.4 | 97.6 | 35.2 |
| FZXmut14 | 54006288 | 16186525058 | 99.4 | 97.5 | 33.5 |
| FZXmut15 | 53853591 | 16139984844 | 99.4 | 97.6 | 33.4 |
| FZXmut16 | 55087131 | 16509921586 | 99.4 | 97.6 | 34.2 |
| FZXmut17 | 55632296 | 16674392458 | 99.4 | 97.7 | 34.5 |
| FZXmut18 | 58724312 | 17599463410 | 99.5 | 97.9 | 36.4 |
| FZXmut19 | 57381979 | 17197700942 | 99.4 | 97.7 | 35.6 |
| FZXmut2 | 54821500 | 16429581486 | 99.4 | 97.7 | 34.0 |
| FZXmut20 | 55926252 | 16758046492 | 99.4 | 97.6 | 34.7 |
| FZXmut3 | 60432008 | 18110588956 | 99.5 | 97.9 | 37.5 |
| FZXmut4 | 57396241 | 17199169358 | 99.4 | 97.6 | 35.6 |
| FZXmut5 | 55408667 | 16606195622 | 99.4 | 97.5 | 34.4 |
| FZXmut6 | 57626027 | 17270339512 | 99.4 | 97.6 | 35.7 |
| FZXmut7 | 56107716 | 16814344566 | 99.4 | 97.6 | 34.8 |
| FZXmut8 | 54289645 | 16272259580 | 99.4 | 97.6 | 33.7 |
| FZXmut9 | 55691946 | 16692044696 | 99.4 | 97.7 | 34.5 |
| Ln15mut1 | 56238421 | 16851692210 | 99.4 | 97.5 | 34.9 |
| Ln15mut10 | 51033247 | 15291987282 | 99.4 | 97.6 | 31.6 |
| Ln15mut11 | 58927164 | 17659804158 | 99.4 | 97.5 | 36.5 |
| Ln15mut12 | 57011463 | 17088015436 | 99.3 | 97.4 | 35.3 |
| Ln15mut13 | 51701332 | 15494699420 | 99.3 | 97.3 | 32.1 |
| Ln15mut14 | 56154466 | 16826760092 | 99.4 | 97.6 | 34.8 |
| Ln15mut15 | 52494044 | 15730608286 | 99.4 | 97.5 | 32.5 |
| Ln15mut16 | 58920074 | 17659795782 | 99.4 | 97.7 | 36.5 |
| Ln15mut17 | 57411377 | 17204950780 | 99.4 | 97.7 | 35.6 |
| Ln15mut18 | 55170929 | 16534019510 | 99.4 | 97.7 | 34.2 |
| Ln15mut19 | 54150380 | 16226469174 | 99.5 | 97.8 | 33.6 |
| Ln15mut2 | 56375232 | 16893504108 | 99.4 | 97.6 | 34.9 |
| Ln15mut20 | 50100547 | 15017663322 | 99.3 | 97.3 | 31.1 |
| Ln15mut3 | 57394712 | 17199333564 | 99.4 | 97.4 | 35.6 |
| Ln15mut4 | 51498983 | 15433704828 | 99.4 | 97.5 | 31.9 |
| Ln15mut5 | 51840698 | 15534322146 | 99.4 | 97.5 | 32.1 |
| Ln15mut6 | 53942857 | 16165049518 | 99.4 | 97.7 | 33.4 |
| Ln15mut7 | 61073658 | 18304384820 | 99.4 | 97.6 | 37.9 |
| Ln15mut8 | 69621829 | 20864431094 | 99.3 | 97.4 | 43.2 |
| Ln15mut9 | 52507826 | 15734796168 | 99.4 | 97.5 | 32.5 |
